# Accounting for missing data in statistical analyses: multiple imputation is not always the answer

**DOI:** 10.1093/ije/dyz032

**Published:** 2019-03-16

**Authors:** Rachael A Hughes, Jon Heron, Jonathan A C Sterne, Kate Tilling

**Affiliations:** 1 Population Health Sciences, Bristol Medical School, University of Bristol, Bristol, UK; 2 MRC Integrative Epidemiology Unit, University of Bristol, Bristol, UK; 3 NIHR Bristol Biomedical Research Centre, University of Bristol, Bristol, UK

**Keywords:** Complete case analysis, inverse probability weighting, missing data, missing data mechanisms, missing data patterns, multiple imputation

## Abstract

**Background:**

Missing data are unavoidable in epidemiological research, potentially leading to bias and loss of precision. Multiple imputation (MI) is widely advocated as an improvement over complete case analysis (CCA). However, contrary to widespread belief, CCA is preferable to MI in some situations.

**Methods:**

We provide guidance on choice of analysis when data are incomplete. Using causal diagrams to depict missingness mechanisms, we describe when CCA will not be biased by missing data and compare MI and CCA, with respect to bias and efficiency, in a range of missing data situations. We illustrate selection of an appropriate method in practice.

**Results:**

For most regression models, CCA gives unbiased results when the chance of being a complete case does not depend on the outcome after taking the covariates into consideration, which includes situations where data are missing not at random. Consequently, there are situations in which CCA analyses are unbiased while MI analyses, assuming missing at random (MAR), are biased. By contrast MI, unlike CCA, is valid for all MAR situations and has the potential to use information contained in the incomplete cases and auxiliary variables to reduce bias and/or improve precision. For this reason, MI was preferred over CCA in our real data example.

**Conclusions:**

Choice of method for dealing with missing data is crucial for validity of conclusions, and should be based on careful consideration of the reasons for the missing data, missing data patterns and the availability of auxiliary information.

**Key Messages**

- When the exposure and/or confounders in the main analysis are missing not at random (MNAR), complete case analysis (CCA) is a valid approach but multiple imputation (MI) may give biased results.
- MI is a valid approach for all missing at random (MAR) mechanisms, whilst CCA may give biased results when the chance of being a complete case depends on the observed values of the outcome.
- Unlike CCA, MI can use information from auxiliary variables (not included in the main analysis) that explain the reasons for missing data and/or provide information about the missing values.
- Efficiency gains of MI over CCA are greatest when there are small amounts of missing data on many variables and/or auxiliary variables that provide information about the missing values.

## Introduction

Failure to appropriately account for missing data in analyses may lead to bias and loss of precision (‘inefficiency’).^1^ Over the past 20 years there has been extensive development of statistical methods^1–3^ and software^4–16^ for analysing data with missing values. Principled methods of accounting for missing data include full information maximum likelihood estimation,^1^^,^^17^^,^^18^ multiple imputation (MI)^1^^,^^19^^,^^20^ and weighting adjustment methods.^21–24^ However, there are circumstances in which a ‘complete case analysis’ (CCA) (an analysis restricted to individuals with complete data) is an appropriate choice;^25–27^ this is not widely known by authors of epidemiological studies. All statistical methods for analysing data with missing values (‘incomplete data’) require assumptions about the reasons for missing data. Choice of method should account for the amount of, patterns of, and reasons for the missing data; no single method is appropriate for all situations.

We review the circumstances in which CCA will not be biased by missing data, describe MI, and compare the bias and efficiency of CCA and MI in a range of missing data scenarios depicted using causal diagrams. We illustrate these scenarios using a hypothetical example, and show how to select an appropriate method for analysing incomplete data using a real data analysis from the Barry Caerphilly Growth Study.^28^^,^^29^

## Illustrative hypothetical example

Our hypothetical example concerns the relationship of cannabis use at age 15 with mental health problems at age 21: the outcomes are depression symptom score (continuous) and whether the participant had deliberately self-harmed within the year before their 21st birthday (‘self-harm’, binary). The exposure of interest is self-reported cannabis use within the past year (none, less than weekly, weekly) measured at age 15. Other variables include maternal substance use (ever tobacco or cannabis use, alcohol use above recommended limits), child’s sex, child depression symptom score (at age 12) and child conduct disorder.

We consider two analyses: linear regression of depression symptom score on cannabis use and logistic regression of self-harm on cannabis use. The models’ covariates are the exposure of interest (cannabis use) and potential confounders child’s sex and maternal substance use behaviours. We refer to variables not included in the main analysis (for example, child conduct disorder) as ‘auxiliary variables’, and we assume that in the absence of missing data the main analysis would give unbiased results.

## Reasons for missing data

Reasons for missing data (known as missingness mechanisms) are commonly classified as ‘missing completely at random’ (MCAR), ‘missing at random’ (MAR), and ‘missing not at random’ (MNAR)^30^ (see Box 1 for definitions and examples). It is not possible to distinguish between MAR and MNAR based only on the observed data: we must generally use our knowledge of the study and subject matter to decide whether MAR is plausible. For example, the possibility that cannabis use is MNAR must be considered if the adolescent participants expressed concern regarding the confidentiality of their self-reported responses to the smoking-related questions. However, exploratory analyses can refute MCAR by identifying observed predictors of the missingness mechanism.^19^ For example, distributions of variables for socioeconomic status can be compared between participants with observed and missing tobacco use.

**Box 1. Definitions of statistical terms**

- Complete Case Analysis (CCA) – An analysis restricted to individuals with complete information on all variables of the main analysis.
- Multiple Imputation (MI) – Missing values are replaced by plausible values (‘imputed values’). To account for uncertainty about the imputed values, multiple such completed datasets are created. These are analysed separately using standard statistical methods and the multiple sets of results combined using ‘Rubin’s rules’.
- Missing Completely At Random (MCAR) – When data are MCAR there are no systematic differences between the observed and missing data: for example if self-reported cannabis use was sometimes not recorded because some adolescents skipped the relevant question due to randomly occurring printer or software errors.
- Missing At Random (MAR) – When data are MAR any systematic differences between the observed and missing data can be explained by associations with the observed data: for example if cannabis use was more likely to be missing among adolescents who smoked weekly but only because they were more likely to come from families with low socio-economic position, who were less likely to attend the clinic visit where cannabis use was measured.
- Missing Not At Random (MNAR) – When the missingness mechanism is neither MCAR nor MAR it is MNAR, in which case associations with the observed data cannot explain all systematic differences between the observed and missing data. For example, if adolescents who had used cannabis were less likely to answer the cannabis-related questions because they were worried this information would be passed on to their parents or teachers, and such concerns could not be explained by measured variables.
- Inverse Probability Weighting – A weighted analysis, in which the complete cases are weighted by the inverse of the probability of being a complete case. The weights are used to try to make the complete cases representative of all cases.

**Figure 1.**
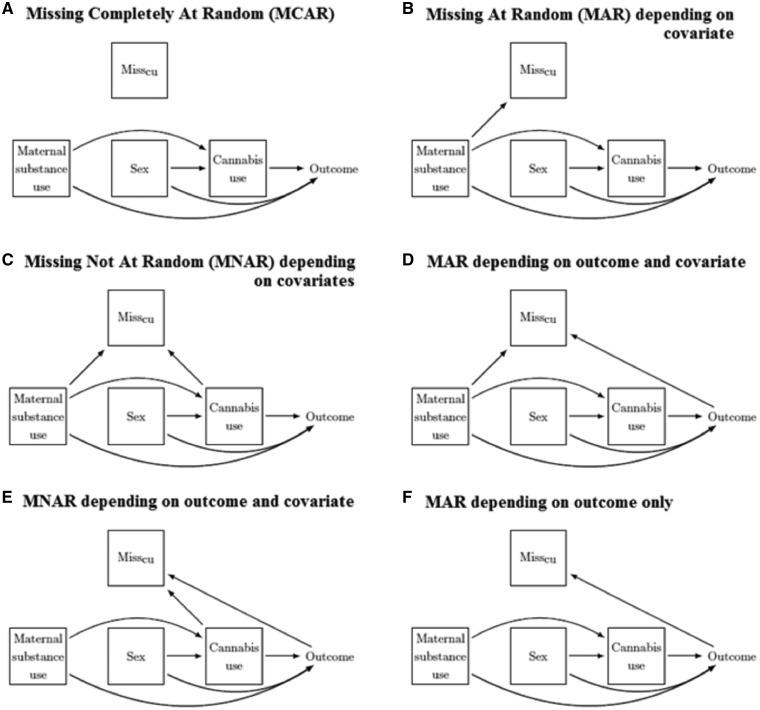
Diagrams showing causal relationships between the completely observed outcomes of the linear and logistic regression (depression symptom score and self-harm respectively), completely observed covariates maternal substance use and sex, incompletely observed exposure cannabis use, and MissCU, a binary variable that indicates whether cannabis use is observed or missing. Note, for clarity we have not included all arrows between the covariates.

Causal diagrams, which depict assumed causal relationships between two or more variables,^31^ can be used to depict assumptions about missingness mechanisms.^32^^,^^33^Figure 1 shows diagrams of six possible mechanisms for a simple scenario where only cannabis use has missing data. A binary variable, Miss_CU_, indicates whether cannabis use is observed or missing. In Figure 1A missingness does not depend on the outcome or the covariates, and so data are MCAR. In Figure 1B and C, missingness does not depend on the outcome: Figure 1B shows an MAR mechanism (missingness depending on maternal substance use) and Figure 1C shows an MNAR mechanism (missingness depending on maternal substance use and the missing values of cannabis use). In Figure 1D–F, missingness depends on the outcome: Figure 1D shows an MAR mechanism (missingness depends on the outcome and maternal substance use), Figure 1E shows an MNAR mechanism (missingness depends on the outcome and the missing values of cannabis use) and Figure 1F shows an MAR mechanism (missingness depends only on the outcome).

## When will missing data lead to bias in a complete case analysis?

Whether a CCA is biased by missing data depends on the missingness mechanism and the type of analysis. Following Bartlett *et al*.,^27^Table 1 summarises the situations in which CCA does and does not lead to bias, for analyses using linear or logistic regression. The Supplementary Material, available as Supplementary data at *IJE* online, contains an extended version of this table. These rules apply regardless of whether the missing values are in the outcome, exposure or confounders.^27^ Since it is impossible to cover all eventualities, there are special cases we have not discussed.

**Table 1.** Potential bias of the exposure regression coefficient in complete case analysis based on linear or logistic regression, according to the reasons for missing data. Unless otherwise stated, the entries apply to both Missing At Random and Missing Not At Random missingness mechanisms

	Exposure regression coefficient	
Variables missingness is dependent upon	Linear	Logistic
None (i.e. Missing Completely At Random)	Unbiased	Unbiased
Outcome	Biased^a^	Unbiased
Exposure (and possibly confounders)	Unbiased	Unbiased
Outcome and confounders	Biased	Unbiased
Outcome and exposure (and possibly confounders)	Biased	Biased^b^

a Biased in general, except when in truth there is no association between the outcome and the exposure (i.e. the true value of the exposure regression coefficient is zero).

b Biased in general, except when missingness depends on the outcome and exposure independently.

CCA is not biased by missing data when the data are MCAR, because the complete cases are representative of those with missing data. Contrary to widespread belief that MCAR is required for CCA to be unbiased,^34^ CCA can give unbiased results in situations where data are MAR or even MNAR.^19^^,^^25^^,^^27^

For most regression models, including linear and logistic regression, CCA also gives unbiased results when the chance of being a complete case does not depend on the outcome after taking the covariates into consideration (for example, by including them in the regression model).^1^^,^^19^

For example, in the MAR mechanism shown in Figure 1B, maternal substance use predicts both the outcome and whether cannabis use is missing, and therefore the chance of being a complete case is associated with the outcome. This means that a CCA that does not include maternal substance use will be biased by the missing data because there is an open path between Miss_CU_ and the outcome via maternal substance use. However, including maternal substance use blocks this path, so that the chance of being a complete case no longer depends on the outcome and the bias is removed. For the same reason, a CCA of the MNAR mechanism shown in Figure 1C is not biased by the missing data because adjusting for maternal substance use and cannabis use blocks all pathways between Miss_CU_ and the outcome.

In general, the results from a CCA using linear regression are biased when the chance of being a complete case depends on the outcome even after taking the covariates into consideration.^19^^,^^25^ The MAR mechanism shown in Figure 1D and F and the MNAR mechanism shown in Figure 1E are examples of such situations.

For logistic regression, there are three additional situations in which a CCA gives an unbiased estimate of the exposure odds ratio,^19^^,^^27^ which arise because the disease odds ratio equals the exposure odds ratio (for example, the same odds ratio is obtained from the logistic regression of self-harm on cannabis use and the logistic regression of cannabis use on self-harm, but missingness depending on self-harm is MAR depending on the outcome for the former, and MAR depending on the exposure for the latter).

1. The chance of being a complete case only depends on the outcome; for example, the MAR mechanism depending only on self-harm shown in Figure 1F.
2. The chance of being a complete case only depends on the outcome and the confounders; for example, the MAR mechanism depending on self-harm and maternal substance use shown in Figure 1D.
3. The chance of being a complete case depends on the outcome and the exposure independently. This would be the case, for example, if the outcome ‘self-harm’ is less likely to be observed among those who did not self-harm (irrespective of cannabis use), and cannabis use is less likely to be observed among those who smoked (irrespective of self-harming).

These exceptions for logistic regression do not apply if the binary outcome is a dichotomized continuous outcome and missingness depends on the underlying continuous outcome; for example, if self-harm is derived by dichotomizing a continuous score measuring the propensity to self-harm and missingness depends on this continuous score.

## Multiple imputation

In this approach, we use an ‘imputation model’ to randomly sample values of the missing data (‘imputed values’) from their predicted distribution based on the observed data. The completed dataset (with the missing values replaced by imputed values) can be analysed using standard statistical methods. Our uncertainty about the missing values is accounted for by creating multiple such datasets. The results from the multiple datasets are combined using ‘Rubin’s rules’, and the standard errors of the estimates of interest properly reflect the uncertainty about the missing values.^1^^,^^19^ The greater the loss of information due to missing data, the greater the variability between the different completed datasets, and the larger the standard errors of the estimates of interest. Most implementations of MI assume data are MCAR or MAR.^25^

Imputation models should contain all the variables in the analysis model (including the outcome), plus variables that predict missingness (in our example, variables that predict whether cannabis use is observed or missing) and variables that predict the values of the incomplete variables (in our example, variables that predict cannabis use). In addition to the main analysis variables, the imputation model usually includes ‘auxiliary’ variables (in our example, child depression symptom score), either because they are associated with missingness or because they are associated with the incomplete variables so that their inclusion improves efficiency. A valuable source of auxiliary data is proxy (or surrogate) data for an incomplete variable.^35–38^ For example, linked data on national exam results at age 16 were used as a proxy for IQ at age 15 years, where IQ was suspected to be MNAR, to increase the plausibility of the MAR assumption and improve precision.^37^^,^^38^ It is important to ensure that the imputation model’s assumptions are plausible and that the assumptions of the imputation model and the main analysis do not conflict with each other (for example, the imputation model must account for any interactions of the main analysis).^19^^,^^39^ Advice is available on building an imputation model (for example,^19^) and on techniques to help determine when the imputed values are reasonable (for example,^40^). MI has been adapted to a variety of different types of data (for example, survival data).^19^^,^^41–43^ Imputation approaches that allow for MNAR mechanisms or perform a sensitivity analysis to departures from MAR have been proposed,^3^^,^^8^^,^^12^^,^^16^^,^^19^^,^^38^^,^^44–49^ but most common implementations of imputation in commercially available software assume MAR.

Several different methods can be used to impute missing values, including joint modelling imputation,^1^^,^^50^ fully conditional specification imputation^51–53^ and hotdeck imputation.^44^^,^^54^ As with any statistical analysis, the approach used should be decided a priori, with the reasons for the selected approach clearly stated in the description of the analysis methods. It is appropriate to check whether the chosen method produces reasonable values of imputed data, but it would not be appropriate to choose a method based on the results of the substantive analysis accounting for missing data.

## Selection of an appropriate missing data method

We now compare MI (assuming MAR) and CCA with respect to bias and efficiency when the analysis model is a linear or logistic regression.

### Bias

MI gives unbiased results for data that are MCAR (such as in Figure 1A) or MAR (such as in Figure 1B, D and F). Further, MI can accommodate situations in which the missingness mechanism depends on auxiliary variables, by including these variables in the imputation model.

In general, MI gives biased results for MNAR mechanisms (such as in Figure 1C and E) because most implementations of MI assume data are MAR given the variables included in the imputation model. Therefore, CCA is preferable for data that are MNAR, in situations such as in Figure 1C, where a CCA gives unbiased results for the estimates of interest. In this situation imputing the missing values of cannabis use would cause bias.

### Efficiency

Auxiliary variables can provide information about the missing values of the main analysis variables and so improve the efficiency of MI. For example, children’s depression symptom score and conduct disorder at age 12 could provide information about their missing cannabis use. Therefore, when information from auxiliary variables is available and the imputation model is correctly specified and does not lead to bias, MI is preferable to CCA.^25^

Where there are no available auxiliary variables, the gain in efficiency from MI depends upon the amount of information about the exposure regression coefficient contained in the incomplete cases, which are discarded by CCA but used by MI. Box 2 discusses the amount of information contained in the incomplete cases, in different situations.

**Box 2. Amount of information about the regression coefficients that the incomplete cases are likely to contain, in the absence of auxiliary variables, for different missing data patterns**

- When **only the outcome variable has missing values** then the incomplete cases do not contain any information about the exposure coefficient or the other coefficients.^1^^,^^19^^,^^55^ In this situation, no information is gained from imputing the outcome. Standard errors from multiple imputation (MI) are likely to be larger than those of complete case analysis (CCA) so that CCA is the best choice.^55^
- When **only the exposure has missing values** then the incomplete cases contain minimal information about the exposure coefficient, although they can contain information about the confounders’ coefficients.^25^ Therefore, CCA is appropriate if interest is only in the exposure. If the other regression coefficients are also of interest, then MI is preferable.^25^
- When individuals tend either to have **observed values for all covariates or missing values for most covariates** (e.g., if missingness only occurs when a particular questionnaire is not filled in) then the incomplete cases are unlikely to contain much information about the regression coefficients,^23^ especially when the number of incomplete variables is large relative to the number of fully observed variables. In this situation, MI results may also be highly susceptible to misspecification of the imputation model.^23^ Therefore, in this situation, CCA may be preferred to MI.
- The incomplete cases are most likely to contain substantial information about the exposure coefficient when there are **many covariates each with small amounts of missing data**, and individuals tend to have missing values on different variables, so that there is a large proportion of incomplete cases.^25^ In this situation, MI can lead to substantial efficiency gains compared to CCA.

## Real data example

We illustrate a missing data analysis of real data from the Barry Caerphilly Growth Study.^29^ This is a follow-up of a dietary intervention randomized controlled trial of pregnant women and their offspring, who were followed up until aged 5 years.^28^^,^^29^ Data were collected on the offspring’s parents (anthropometric measures, health behaviours and socioeconomic characteristics) and the offspring (gestational age, sex, and 14 weight and height measures at birth, 6 weeks, 3, 6, 9 and 12 months, and thereafter at 6-monthly intervals). When aged 25, these offspring were invited to participate in a follow-up study in which standard anthropometric measures were recorded. We refer to the offspring, later young adults in the follow-up study, as the study participants.

Our main analysis was a linear regression of adult body mass index (BMI) (at age 25) on weight at age 5. Other covariates were birth weight, sex, gestational age, maternal weight, paternal weight and parental socioeconomic status in childhood. Among the 951 participants, birth weight and sex were completely observed, whereas adult BMI, paternal weight, gestational age, weight at age 5, parental socioeconomic status and maternal weight were missing for, respectively, 272, 141, 45, 8, 3 and 1 participants. Of the 679 participants with observed adult BMI 2.21% were underweight (BMI < 18.5 kg/m^2^), 53.31% were normal weight (18.5 kg/m^2^ ≤ BMI < 25 kg/m^2^), 31.66% were overweight (25 kg/m^2^ ≤ BMI < 30 kg/m^2^) and 12.81% were obese (BMI ≥ 30 kg/m^2^), which is similar to the distribution of BMI among adults living in the UK.^56^

**Figure 2.**
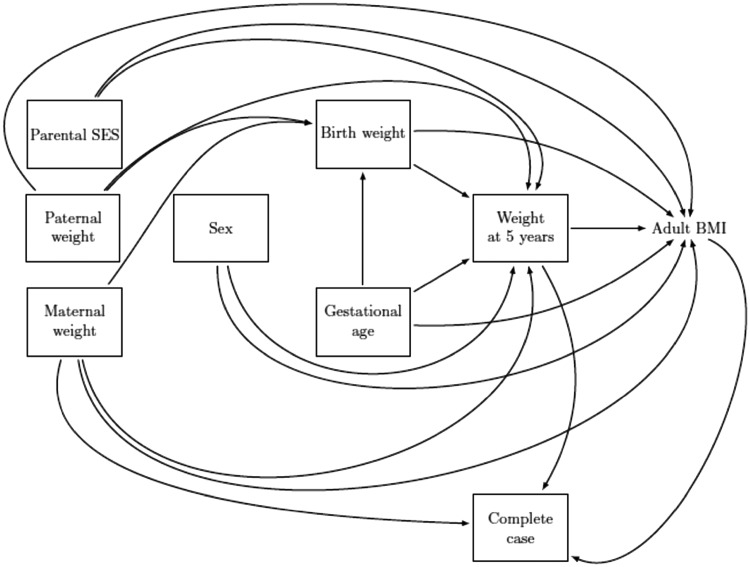
Diagram showing the causal relationship between the outcome [adult body mass index (BMI), exposure (weight at age 5), confounders (birth weight, sex, gestational age, maternal weight, paternal weight and parental socioeconomic status (SES)], and complete case, a binary variable that indicates whether a participant is a complete case (observed values for the outcome, exposure and all confounders) or an incomplete case (missing values for at least one of these variables). Note, we have not included all arrows between the covariates.

First, we investigated whether the chance of being a complete case depends on the outcome after conditioning on the main analysis covariates. See the Supplementary Material, available as Supplementary data at *IJE* online, for more details. Table 2 shows that the chance of being a complete case was associated with the observed values of the outcome (adult BMI), the exposure (weight at 5 years) and maternal weight. This is shown in Figure 2, which is a causal diagram depicting assumed relationships between the outcome, the covariates of the main analysis and being a complete case. These relationships correspond to more general version of the MAR mechanism (missingness depending on the outcome and covariates) shown in Figure 1D. We therefore concluded that a CCA of these data was likely to give biased results.

**Table 2.** Results of the missingness model applied to 679 participants with observed values for adult body mass index (BMI), weight at 5 years and maternal weight

	Odds ratio	95% CI
Weight at 5 years (kg) (exposure variable)	0.913	0.827, 1.01
Birth weight (kg)	1.19	0.775, 1.83
Sex	0.721	0.479, 1.09
Maternal weight (kg)	0.950	0.924, 0.976
Adult BMI (kg/m^2^) (outcome variable)	1.06	1.01, 1.11

CI, confidence interval.

Further investigations revealed that a subset of the childhood height and weight measurements predicted missingness in adult BMI, gestational age and paternal weight, with the remaining variables having too few missing values to be able to detect any observed predictors of missingness.

We next examined missing data patterns for the main analysis variables, to establish whether the incomplete cases contained information about the exposure coefficient (weight at age 5). Table 3 shows that there were 404 incomplete cases, of which 272 were missing the outcome, adult BMI (patterns 4–6). Among these 272 cases, 210 had complete data for the exposure and confounders and the remaining 62 had some observed data on the exposure and confounders. Therefore, these 272 cases with a missing outcome could reduce uncertainty when imputing the values of missing covariates in the 132 incomplete cases with an observed outcome (patterns 2–3). Also, the 125 incomplete cases with an observed outcome and observed exposure (pattern 2) were likely to contain information about the exposure coefficient. We concluded that a substantial proportion of the incomplete cases contained information that could be utilized by MI to improve efficiency.

**Table 3.** Missing data patterns of the main analysis variables: outcome (adult BMI), exposure (weight at 5 years), confounders (maternal weight, paternal weight and parental socioeconomic status) for 951 participants of the Barry Caerphilly Growth Study. ✓ denotes observed, × denotes missing, and ✓/× denotes some observed and some missing. Omitted variables sex and birth weight were completely observed

	Follow-up study	Original childhood study		
Pattern	Outcome	Exposure	Confounders	Number of participants (%)
1	✓	✓	✓	547 (57.5%)
2	✓	✓	✓/×	125 (13.1%)
3	✓	×	✓	7 (0.7%)
4	×	✓	✓	210 (22.1%)
5	×	✓	✓/×	61 (6.4%)
6	×	×	✓	1 (0.1%)

These analyses led us to choose MI over CCA because: (i) a CCA could produce biased results because the chance of being a complete case depended on the outcome, (ii) there were auxiliary variables that predicted missingness, (iii) the incomplete cases were likely to contain information that could be utilized by MI, (iv) there were auxiliary variables (childhood height and weight measurements, maternal and paternal height) that predicted the missing values of the outcome, exposure and confounders and (v) there was sufficient observed information to construct an appropriate imputation model.

We used chained equations imputation (also known as fully conditional specification)^6^^,^^57^ that can handle different types of variables since each variable is imputed using its own regression model. For each incomplete variable, we included the other variables of the main analysis, and auxiliary variables as predictors of its regression model. We conducted MI with 50 imputations under the assumption data were MAR. The plausibility of the MAR assumption is discussed in the Supplementary Material, available as Supplementary data at *IJE* online.

Table 4 shows the results of the main analysis using CCA and using MI, with estimated associations shown as log odds ratios to facilitate comparison between standard errors for the two approaches. The estimated exposure coefficient, weight at age 5, was similar between the two approaches. However, there were noticeable differences in the estimated coefficients for birth weight, gestational age and parental socioeconomic class. For all covariates, including the exposure, the standard errors were smaller for MI, demonstrating the efficiency gain of MI over CCA.

**Table 4.** Results of complete case and multiple imputation analyses of the association of weight at 5 years with adult BMI, using data from the Barry Caerphilly Growth Study

		Complete case analysis (*n* = 547)			Multiple imputation (*n* = 951; *m* = 50)		
		Log OR	SE	95% CI	Log OR	SE	95% CI
Weight at 5 years (kg) (exposure variable)		0.467	0.0876	0.295, 0.639	0.458	0.0735	0.314, 0.602
Birth weight (kg)		–0.176	0.438	–1.04, 0.684	–0.788	0.410	–1.60, 0.0200
Sex		0.209	0.372	–0.521, 0.940	0.165	0.334	–0.492, 0.822
Gestational age:		0.635	0.610	–0.564, 1.83	0.150	0.565	–0.963, 1.26
39–40 weeks	< 39 weeks	0 (reference)			0 (reference)		
> 41 weeks		–0.00779	0.561	–1.11, 1.09	0.321	0.476	–0.615, 1.26
Maternal weight (kg)		0.0810	0.0198	0.0421, 0.120	0.0835	0.0183	0.0475, 0.120
Paternal weight (kg)		0.0463	0.0180	0.0110, 0.0816	0.0477	0.0170	0.0143, 0.0812
Parental socioeconomic status:	I/II	–0.633	0.493	–1.60, 0.334	–0.791	0.453	–1.68, 0.101
III		0 (reference)			0 (reference)		
IV/V		1.07	0.465	0.158, 1.99	1.20	0.449	0.317, 2.09

*n,* number of observations; *m*, number of imputations; log OR, odds ratio on the natural logarithm scale; SE, standard error; CI, confidence interval.

## Final remarks

Missing data is a pervasive problem that should be dealt with appropriately. Transparent reporting of how missing data could affect the results of the main analysis is crucial.^58^ It is important to conduct sensitivity analyses to the assumptions made about the missing data and any other assumptions relevant to the method used.^1^^,^^19^^,^^23^ There may also be concerns specific to the type of study being analysed: for example, respecting the intention to treat principle in randomized trials.^59^

We have considered bias of a CCA when the analysis is a linear or logistic regression model. For most regression models, including probit regression and Cox proportional hazards regression for time-to event outcomes, a CCA is not biased by missing data when the chance of being a complete case does not depend on the outcome.^19^^,^^27^^,^^55^ Furthermore, for Cox regression, the situations in which the exposure coefficient is not biased by missing data are the same as those for logistic regression, providing that follow-up is the same across participants and the event rate is low.^27^

Valid results from MI depends on careful construction of the imputation model. Therefore, it is important that researchers check that the assumptions of the imputation model are plausible^60–62^ and examine the sensitivity of the results to any assumptions that cannot be verified from the observed data.^48^ The potential for misspecification of the imputation model depends on several factors including the complexity of the analysis of interest, types of variables to be imputed and the missing data pattern. MI results are likely to be more susceptible to misspecification of the imputation model as the amount of missingness increases.^63^ Consequently, researchers often ask if there is an upper threshold on how much data can be imputed. Some studies have shown MI to be beneficial even for large proportions of missing data: for example, an outcome of linear regression with up to 90% of data MAR imputed using auxiliary information,^64^ a confounder of linear regression with up to 90% of data MAR,^63^ and skewed continuous covariates of a Cox proportional hazards regression with up to 50% of data MAR.^65^ It is impossible to specify an upper threshold for the proportion of missing values since the potential benefits of MI over CCA depend on many factors including the missing data mechanism, missing data pattern, availability of auxiliary variables and feasibility of correctly specifying the imputation model.^63^

Inverse probability weighting is an alternative to MI that can be advantageous when misspecification of the imputation model is likely.^23^ The Supplementary Material, available as Supplementary data at *IJE* online, and Perkins *et al*.^66^ describe the advantages and disadvantages of inverse probability weighting compared with MI.

In summary, CCA can be a better alternative to MI for the analysis of data with missing values in certain situations. However, when auxiliary variables are available (providing information about the missing values and improving the plausibility of MAR) then MI is often the preferred choice over CCA. Choice of method should be based on careful consideration of the nature of the main analysis, the reasons for missing data, missing data patterns, availability of auxiliary information and the feasibility of implementing the method correctly.

## Funding

R.A.H. was supported by Medical Research Council grant [MR/J013773/1]. J.H. was supported by the Medical Research Council and Alcohol Research UK grant [MR/L022206/1]. J.A.C.S. was supported by National Institute for Health Research Senior Investigator award NF-SI-0611–10168. R.A.H. and K.T. were supported by the Medical Research Council Integrative Epidemiology Unit at the University of Bristol (MC_UU_00011/3). J.H., J.A.C.S. and K.T. were supported by the National Institute for Health Research Bristol Biomedical Research Centre.

## Supplementary Material

dyz032_Supplementary_Data
