# Supplementary material for: Accounting for missing data in statistical analyses: multiple imputation is not always the answer
Source: Int J Epidemiol. 2019 Mar 16;48(4):1294–304. doi: 10.1093/ije/dyz032 (PMC6693809; doi:10.1093/ije/dyz032)
Supplement: dyz032_Supplementary_Data [file dyz032_supplementary_data.docx]

**Accounting for missing data in statistical analyses: multiple imputation is not always the answer**

**Supplementary material**

**Real data analysis**

Investigation of the missingness mechanisms

We investigated whether the chance of being a complete case depends on the outcome after conditioning on the main analysis covariates. We used a logistic regression model, which we refer to as the “missingness model”, in which the outcome was a binary variable, indicating whether the participant had complete or incomplete data on the variables in the analysis model. We fitted the missingness model to the observed data, and the predictors of the model were a subset of the main analysis variables, including adult BMI. We could not include all of the main analysis variables as predictors because then the model would only be fitted to those participants with complete data for these variables, and so the binary outcome (complete or incomplete data) would not vary. Instead, we selected the predictors using a series of logistic regression models [1]. First, for each incomplete variable in turn, we fitted a missingness model including the said incomplete variable and all completely observed variables (birth weight and sex) as predictors. Second, we fitted a missingness model where the predictors were birth weight, sex and adult BMI, and the variables identified from the first stage as predictive of being a complete case. From these investigations we concluded that the chance of being a complete case was associated with the observed values of the outcome (adult BMI), the exposure (weight at 5 years) and maternal weight (see Table 2 of the main paper).

A similar approach was used to identify predictors of the missingness mechanism for a specific variable (such as paternal weight) by setting the dependent variable of the missingness model to be a binary variable indicating whether that variable was observed or missing. Here, we also included auxiliary variables (for example, childhood height measurements) as predictors of the missingness model. From our investigations a subset of the childhood height and weight measurements predicted missingness in the following variables: outcome (adult BMI), gestational age, and paternal weight. For the remaining variables, which have at most 1.1% missing data, we were unable to detect any observed predictors of missingness.

Plausibility of the missing at random assumption

The imputation approach we used assumed the data were MAR. Participants missing the outcome, adult BMI, did not attend the follow-up clinic. It is plausible that reasons for non-attendance were related to their unobserved BMI measurement (for example, if a participant did not attend because she or he was uncomfortable with her/his body size). However, since

weight and height during childhood predicted missingness and were associated with adult BMI then adding these auxiliary variables to the imputation model increases the plausibility that adult BMI was MAR. Similarly, for gestational age and paternal weight. The remaining covariates (weight at age 5, parental socioeconomic status and maternal weight) had small amounts of missing data. Therefore, even if these covariates were MNAR depending on their missing values, this was unlikely to have a large impact on the results [2].

**Inverse Probability Weighting**

Inverse probability weighting is a type of weighted complete case analysis [3,4] in which the weights are used to try to make the complete cases representative of all cases. The weights are derived from a model for the probability that an individual is a complete case (a “missingness model”). The main analysis model, which is fitted to the complete cases, is weighted by the inverse of these probabilities (the incomplete cases and their weights are discarded). Standard errors are calculated using a sandwich estimator [5, 6]. The efficiency of inverse probability weighting can be improved by including auxiliary variables that are associated with the variables of the main analysis, even when they do not predict missingness [3, 7]. Guidance on how to build a missingness model is available [3, 8]. Goodness of fit tests for logistic regression models can be used to evaluate the missingness model [9, 10].

Like multiple imputation, inverse probability weighting assumes data are MAR [3]. The variables of the missingness model are usually fully observed or have a “monotone” missingness pattern [3].

Comparison of multiple imputation and inverse probability weighting

Results from multiple imputation and inverse probability weighting will be unbiased provided that the data are MAR given the variables of the imputation model and missingness model respectively, and that these models are correctly specified (i.e. their assumptions hold true).

Multiple imputation has two major advantages over inverse probability weighting. First, it is generally more efficient because it uses all observed data from the incomplete cases, whereas inverse probability weighting discards the incomplete cases. Augmented IPW is an extension to IPW which utilises the data from the incomplete cases and hence may improve efficiency over standard implementations of IPW [3; 11]. However, augmented IPW is more complicated to apply in standard software [3]. Second, auxiliary variables of the imputation model can also be incompletely observed whilst inverse probability weighting requires either that all variables of the missingness model are fully observed or that they have a “monotone” missing data pattern (more complicated inverse probability weighting methods may overcome these problems [12-14]).

Inverse probability weighting has three major advantages over multiple imputation. First, it is easier to understand than multiple imputation, particularly for those familiar with sampling weights used in survey analyses. Second, it is generally easier to specify a missingness model than an imputation model, especially when the main analysis contains features, such as nonlinearities, which are difficult to correctly incorporate into the imputation model. Third, inverse probability weighting may be preferable to multiple imputation for certain missingness patterns: for example, when there are large blocks of missing data such that individuals tend either to have observed values for all variables or missing values for the majority of the variables. This may occur, for example, if missingness only occurs when a particular questionnaire is not filled in. For such a missingness pattern the incomplete cases may contain little information about the estimates of the main analysis [3, 15], especially when the number of incompletely observed variables is large relative to the number of fully observed variables. One may also feel uneasy about imputing large blocks of missing data based on a few fully observed variables.

**Supplementary references**

1. White IR, Royston P, Wood AM. Multiple imputation using chained equations: Issues and guidance for practice. Stat Med 2011;30:377–399.
2. Carpenter J.R., Kenward M.G. Multiple imputation and its application. Chichester, West Sussex, UK: Wiley, 2013.
3. Seaman SR, White IR. Review of inverse probability weighting for dealing with missing data. Stat Methods Med Res 2013;22:278–295.
4. Mansournia MA, Altman DG. Inverse probability weighting. BMJ 2016;352:i189.
5. Huber PJ. The behavior of maximum likelihood estimates under nonstandard conditions. In Proceedings of the Fifth Berkeley Symposium on Mathematical Statistics and Probability. Berkeley, Carlifornia, USA: University of California Press, 1967: 1; 221–233.
6. White H. A heteroskedasticity-consistent covariance matrix estimator and a direct test for heteroskedasticity. Econometrica 1980;48:817–830.
7. Tsiatis AA. Semiparametric theory and missing data*.* New York, USA: Springer, 2006.
8. Carpenter JR, Kenward MG, Vansteelandt S. A comparison of multiple imputation and doubly robust estimation for analyses with missing data. J R Stat Soc A 2006;169:571–584.
9. Hinkley D. Transformation diagnostics for linear models. Biometrika 1985;72:487–496.
10. Hosmer DW, Lemeshow S. Applied logistic regression. New York, USA: Wiley, 1989.
11. Vansteelandt S, Carpenter J, Kenward MG. Analysis of incomplete data using inverse probability weighting and doubly robust estimators. Methodology 2010;6:37–48.
12. Robins JM and Gill RD. Non-response models for the analysis of non-monotone ignorable missing data. Stat Med 1997;16:39–56.
13. Seaman SR, White IR. Inverse Probability Weighting with Missing Predictors of Treatment Assignment or Missingness. Commun Stat-Theor M 2014:43;3499–3515.
14. Sun B, Perkins NJ, Cole SR, Harel O, et al. Inverse probability-weighted estimation for monotone and nonmonotone missing data. Am J Epidemiol 2018;187:585–591.
15. Seaman SR, White IR, Copas AJ, Li L. Combining Multiple Imputation and Inverse-Probability Weighting. Biometrics 2012; 68:129–137.

Supplementary table 1: Potential bias of the intercept regression coefficient, exposure regression coefficient and confounder regression coefficients, in complete case analysis based on linear or logistic regression, according to the reasons for missing data. Unless otherwise stated, the entries apply to both Missing At Random and Missing Not At Random missingness mechanisms.

|  | Linear regression | | |  | Logistic regression | | |
| --- | --- | --- | --- | --- | --- | --- | --- |
| Variables missingness is dependent upon | Intercept’s  coefficient | Exposure’s  coefficient | Confounders’  coefficients |  | Intercept’s  coefficient | Exposure’s  coefficient | Confounders’  coefficients |
| None (i.e. missing completely at random) | unbiased | unbiased | unbiased |  | unbiased | unbiased | unbiased |
| Outcome | biased | biased^a^ | biased^a^ |  | biased | unbiased | unbiased |
| Exposure and/or other covariates | unbiased | unbiased | unbiased |  | unbiased | unbiased | unbiased |
| Outcome and confounders | biased | biased | biased |  | biased | unbiased | biased |
| Outcome, exposure, and possibly other covariates | biased | biased | biased |  | biased | biased^b^ | biased |

^a^: Biased in general, except when in truth there is no association between the outcome and the exposure or confounder in question (i.e., the true value of the regression coefficient is zero). ^b^: Biased in general, except when missingness depends on the outcome and exposure independently.
